# Des Cals Difformes Et Des Operations Qu’ils Reclament

**Published:** 1842-07-09

**Authors:** 


### MEDICAL EXAMINER.

NEW SERIES.

No. 28.] PHILADELPHIA, JULY 9, 1842. [Vol. I.

### BIBLIOGRAPHICAL NOTICE.

*Des Cals Difformes et des Operations qu'ils reclament.* Par S. Laugier. Paris, 1841. pp. 98.

*On Deformed Callus, &c:* By S. Laugier. Paris, 1841.

This essay is one of the several theses of the late *concours* for the chair of Operative Surgery at the Faculty of Paris. The process of reproduction in bone has at all times excited much interest from its intrinsic practical importance; and much diversity of opinion, respecting the essential nature of the action, necessarily resulted. The ancients believed in the simple exudation of a viscid fluid on the fractured surfaces, which glued them together. Haller supported this view on the authority of the experiments of his pupil Detlef. Hunter taught the organization of the extravasated blood, and the subsequent production from it of the callus. Howship developed his ideas, and appealed to experiment for their confirmation. Duhamel-Dumonceau gave to the periosteum the property of secreting the callus; and his theory was adopted and extended by Dupuytren, whose experiments and pathological observations led him to teach two distinct periods in the reparation of fractured osseous surfaces. 1st. The formation by the periosteum, (as he believed,) and in some instances also by the surrounding soft parts, of an osseous ferrule, as well as of an internal plug, which occupies forty to fifty days in its formation, and which is *provisional;* absorption in it commencing about the fourth or fifth month, at which period direct union had occurred between the fractured ends. This secondary or *definitive* callus was completed about the eighth month, and the surrounding tissues returned to their natural condition. Bordenave, Bichat and Richerand, believed the process to be analogous to that which takes place in union of the soft parts, and that the osseous surfaces united by granulations. Camper described a double callus—one external resulting from the ossification of the souperiosteal gelatine; the other internal, and formed by the separation and lengthening of the internal osseous laminæ, or the expansion of the compact tissue of the bone. Troja saw the ends of the bones during the first days covered by a gelatinous matter, which became cartilaginous, and finally osseous. He noticed the tumefaction of the periosteum up to a certain period, when it became thinner; an internal ossification filled the medullary cavity near the fracture, as well as an external ossification, which was constant. Messrs. Breschet and Villermé undertook an extended series of observations on this vexed question; they observed the whole process of reparation, throughout all its stages, and showed that the discordant results obtained by previous experimenters, were due to the imperfect and partial manner in which they had studied the phenomena.

The subject recently has been investigated by Miescher, and much elucidated by him.* The process he describes as follows: Immediately on the receipt of the fracture, inflammation of the surrounding soft parts—the cellular tissue, periosteum and muscles—occurs, glueing them together, and forming thus a dense capsule around the point of injury. Now, between this capsule and the bone a semi-fluid substance is exuded, which becomes gradually more consistent and very vascular. A similar action goes on at the same time in the medullary canal, and the effused masses subsequently coalesce, forming the substantia intermedia, which resembles fibrous tissue, whilst the muscles, periosteum and cellular tissue, return to their normal state. The inflammatory action in the bone takes place at a period subsequent to its appearance in the soft parts, and commences at that point of the fractured extremities still surrounded by periosteum, both interiorly and exteriorly. The fibrous substance becomes now converted into cartilage, and then bone. According to Miescher, the callus always arises from the bone itself, and never from the periosteum; this last merely supplying the requisite conditions to the bone for the production of the callus—vessels essential to the formative and nutritive process. Ossification commences on the surface of the capsule next to the bone, and extends from this point. The callus contains the peculiar cartilaginous corpuscles, and when ossified assumes the cellular texture of bone. The periosteum, as originally shown by Detlef, and since by Miescher, is formed subsequently to the formation of the callus on its external rugged surface. The views of Miescher have been confirmed by Sir Bransby Cooper, who, at the suggestion of the late Sir Astley Cooper, undertook about the same time, (1835,–'36,) a series of experiments to determine the phenomena which occur in the reparation of fractured bones. In support of the osseous system alone being the source of the bony deposit, he adduces the fact of bones which have an inferior organization, such as the bones of the cranium and the neck of the thigh bone, of being but slowly and imperfectly reproduced. The subject is yet open, we think, to observation and experiment, and will no doubt receive further attention and study under the new laws of development recently promulgated.

The consolidation of fractures is influenced by a variety of circumstances, both in point of time and in the perfection of the process. It takes place

*De inflam. ossium, etc. Ber. 1836. Traite de Pathologie Externe, &c. par Vidal de Cassis. Paris, 1839, p. 13. Muller's Physiology, vol, i., p. 407.

more rapidly in children than in old age; Delamotte saw fractures of the humerus perfectly united by the twelfth day in young infants; and Mr. J. Cloquet mentions the case of a fractured clavicle in a young girl of six years, where there was complete union in nine days. The actual state of health of the patient influences union; and the supervention of acute disease not only arrests the formation of callus, but causes not unfrequently its partial or total absorption. Our author saw the newly formed callus completely absorbed in a healthy individual attacked with variola. The size of the bone also is to be considered, it taking a longer period for the formation of callus in a fractured femur than in a bone of smaller size, as the humerus or clavicle. The duration of the time that the callus is susceptible to external impressions has been variously estimated; and the practical importance of the question makes it important that it should be ascertained. It has been proved that, from the fiftieth day to the sixth or seventh month, the callus has been broken by mechanical means without the bone being injured.

If the coaptation between the fragments is not perfect the provisional callus is more voluminous, and the immediate union of the osseous surfaces is retarded, or does not occur, and the provisional callus becomes itself definitive; a tumour exists at the seat of fracture, depending on the manner of consolidation of the fragments, and from this results the *deformed* or *vicious callus*, by which the form, direction, and length of the bones are altered, and their functions materially impaired. This condition depends on several causes.

1. *On the situation of the fracture.* This occurs where the injury is in the osseous tutamina of cavities, and where reduction is frequently contra-indicated, as well as in the long bones, where all our retentive efforts are unavailing to prevent displacement of the ends, as in fractures of the neck of the thigh bone and the clavicle.

2. *The penetration of the fracture into an articulation.* The difficulty here is in the employment of means to maintain the coaptation of the fragments, and considerable deviation in the direction of the articulation and of motion occurs. In a case of fracture of the anatomical neck of the humerus in the Dupuytren Museum at Paris, the superior fragment has turned on its own axis, so that the articular surface looks entirely forward, and the edges rest on on the glenoid cavity, and the other upon the extremity of the inferior fragment.*

3. *The obliquity of the fracture in a direction favourable to displacement.* These oblique fractures occur most frequently in the neighbourhood

* A case somewhat similar occurred recently in the practice of Mr. Adams, of Dublin, and the specimen is preserved in the Cabinet of the Pathological Society of that city. The great tuberosity was inclined outwards, and formed a considerable curve with the shaft of the bone. See Medical Examiner, vol. i., N. S., p. 363.

of the ankle joint, and favour the dislocation of the foot backwards. In a fracture of the malleoli from above downwards, and from behind forwards, or a similar fracture of the internal malleolus with that of the inferior part of fibula, there would be a prominence forwards of the inferior extremity of the tibia, which no apparatus could prevent, and an irremediable deformity would result. Mr. Laugier mentions a case of fracture through the tibio-tarsal articulation, then in the Hôpital Beaujon, in which there was actual separation of the tibia and fibula by a fragment of the astragalus. The sole of the foot was inclined inwards, so that its external border was sensibly removed from the external malleolus, which was thrown outwards, and by pressure had caused much irritation of the skin over it.

4. *The relative disposition of the fragments.* This occurs most frequently in fractures of the leg, where an intermediate fragment of the tibia is so jammed between the two other fragments that it is impossible to reduce it, whether there be an external wound or not. In very oblique fractures of the tibia, where the pointed extremity of the upper fragment threatens to pierce the integument, though this accident may be prevented by forcible extension and direct pressure in the direction of the bone, a very acute angle will generally remain after consolidation has taken place. Other causes may be enumerated, such as the adoption of an imperfect apparatus, carelessness in the treatment, the indocility of the patient, as well as involuntary movements on his part, and lastly, the too early discontinuance of the apparatus.

To determine what degree of deformity of the callus requires an operation is a delicate question, and in this affection too many operations of *complaisance* are performed. No specific rules to guide the surgeon on this point have yet been laid down by authors, and Mr. Laugier offers the following, which seem to us sound.

"1. Do not interfere with those cases where a cure without slight deformity is impossible or difficult, as in fractures of the clavicle, neck of the femur, of the cotyloid cavity, and of the pelvis.

"2. Let the usefulness of the part be taken into consideration: thus it will be necessary to rupture, for a slight deformity, a vicious callus of the inferior extremity of the radius, of the fibula, and to abstain from interfering for an equal deformity in a part of less importance.

"3. It is well, Avicenna advises it, and Dupuytren insists on it as indispensable, to take into consideration the displacement of the fragments, and the causes which have produced and maintained it. But, adds Dupuytren, the want of mobility, the engorgement of the soft parts, the kind of crust, often thick, under which the fractured surfaces are hid, render this research difficult. The question remains pretty much unsettled. It should be stated as follows: 'A deformed callus being given to determine by the inspection of the limb the relative position of the fragments.'"

In the majority of cases we are enabled to state with certainty the position of the fragments in deformed callus, if we know the displacements they are subject to at any given point, and the causes which effect them.

The next question to be considered is, what are the means by which we are to remedy the deformity and inconveniences arising from deformed callus? The age of the callus is a contra-indication of the first importance, for to effect redressment, the patient must be subjected to a formidable operation, which nothing but imperative necessity would sanction. The ancient surgeons recommended before redressment the employment of external applications, for the purpose of softening the callus previous to the operation. Dupuytren never redressed a deformed callus without previously enveloping the limb in emollient cataplasms, and ordering general and local baths. He even was in the habit of refusing baths to patients who were cured of a recent fracture, fearing that they would soften the callus. The various methods proposed by surgeons for the rectification of deformed callus are fully discussed by our author, as well as in the classical treatises, and to his essay we refer our readers, having already trespassed both on their patience and the limits assigned us. M. C.

